# PRDM5 promotes the proliferation and invasion of murine melanoma cells through up‐regulating JNK expression

**DOI:** 10.1002/cam4.846

**Published:** 2016-08-03

**Authors:** Lei Wang, Qiong‐Qiong Ding, Shan‐Shan Gao, Hai‐Jie Yang, Mian Wang, Yu Shi, Bin‐Feng Cheng, Jia‐Jia Bi, Zhi‐Wei Feng

**Affiliations:** ^1^ College of Life Science and TechnologyXinxiang Medical UniversityXinxiangChina

**Keywords:** JNK, melanoma, metastasis, PRDM5, proliferation

## Abstract

PRDM (PRDI‐BF1 and RIZ domain‐containing) proteins constitute a family of zinc finger proteins and play important roles in multiple cellular processes by acting as epigenetic modifiers. PRDM5 is a recently identified member of the PRDM family and may function as a tumor suppressor in several types of cancer. However, the role of PRDM5 in murine melanoma remains largely unknown. In our study, effect of PRDM5 on murine melanoma cells was determined and results showed that PRDM5 overexpression significantly promoted proliferation, migration, and invasion of murine melanoma B16F10 cells. Consistently, silencing of PRDM5 expression significantly inhibited proliferation, invasion, and migration of B16F10 cells. In vivo study also showed that PRDM5 silencing significantly inhibited the growth and metastasis of melanoma in mice. PRDM5 was then found to increase the expression and activation of JNK in B16F10 cells. JNK silencing significantly reduced PRDM5‐mediated up‐regulation of JNK expression and blocked the PRDM5‐induced proliferation and invasion of B16F10 cells. To further verify the involvement of JNK signaling in PRDM5‐induced progression of B16F10 cells, a specific JNK inhibitor was employed to inhibit the JNK signaling pathway, and results showed that PRDM5‐induced proliferation and invasion of B16F10 cells were abolished. We conclude that PRDM5 promotes the proliferation and invasion of murine melanoma cells through up‐regulating JNK expression and strategies targeting PRDM5 may be promising for the therapy of melanoma.

## Introduction

PRDM (PRDI‐BF1 and RIZ domain‐containing) proteins constitute a family of transcriptional regulators and play important roles during cell differentiation and malignant transformation 1, 2. The human PRDM family consists of 17 known members harboring an evolutionarily conserved N‐terminal PR (PRDI‐BF1‐RIZ1 homologous) domain, followed by variable numbers of C2‐H2 zinc finger repeats which are involved in protein–DNA and protein–protein interactions 3, 4. The PR domain shares high homology with the histone methyltransferases’ SET (Suvar3–9, Enhancer‐of zeste, Trithorax) domain which is a histone methyltransferase (HMTase) catalytic module functioning in the chromatin‐mediated transcriptional regulation 5. PRDM family members are deregulated in a broad spectrum of cancers, acting as either tumor suppressors or oncogenes. For example, PRDM2 (RIZ1) has been reported to have tumor‐suppressive activities in a variety of tumors, and its expression is down‐regulated in the breast cancer and liver cancer due to its promoter methylation. Moreover, PRDM2 overexpression induces cell cycle arrest at G2‐M phase and apoptosis of breast cancer, liver cancer, and colon cancer cells 6, 7, 8, 9. However, PRDM14, another member of PRDM family, acts as an oncogenic transcriptional activator. PRDM14 expression is up‐regulated in lymphoma, leukemia, lymphoid neoplasms, non‐small cell lung cancer, as well as in early stages of breast cancer. As a result, PRDM14 overexpression in breast cancer cell lines promotes cell growth and stimulates the expression of several breast cancer‐promoting genes 10, 11, 12, 13. Therefore, it is worthwhile to ascertain the specific function of each PRDM member under various cancer contexts.

PRDM5, also named PFM2, was first identified from an EST database based on its similarity to PRDM2 14. In zebra fish, PRDM5 plays an essential role in the embryonic convergent extension movements via Wnt signaling pathway 3. In mouse embryonic stem (mES) cells, PRDM5 directly targets the genomic regions involved in early embryonic development and may affect the expression of a subset of developmental regulators during cell differentiation 15. PRDM5 is also a major transcriptional regulator of extracellular matrix genes involved in osteogenesis 16. Several studies have shown that PRDM5 is mutated or silenced in pathological conditions. Rare mutations in PRDM5 have been identified in Brittle cornea syndrome (BCS), a connective tissue disease characterized by thinning of the cornea, whereas it has also been reported that PRDM5 is silenced due to the promoter hypermethylation in breast cancer, liver cancer, lung cancer, ovarian cancer, cervical cancer, and gastrointestinal cancer 17, 18, 19, 20, 21. Furthermore, PRDM5 overexpression may induce cell cycle arrest at G2/M phase and apoptotic cell death in MDAH2774 and BG1 ovarian cancer cell lines, MB435 breast cancer cells, and Ha22T hepatocellular carcinoma cells 14. However, in a pilot study, our results showed that disruption of PRDM5 expression by insertional mutagenesis mediated by pDisrup 8, a specifically designed retroviral vector, reduced the migration potential of murine melanoma B16F10 cells (unpubl. data) and PRDM5 could exert effects in melanoma cells distinct from those in other cell types, but the specific mechanism is needed to be further studied.

In this study, the roles of PRDM5 in the proliferation, migration, and invasion of murine melanoma cells were investigated, and results showed that PRDM5 overexpression significantly enhanced the proliferation, migration, and invasion of murine melanoma B16F10 cells, and PRDM5 silencing reduced their growth and metastasis. In vivo experiments revealed that PRDM5 silencing greatly inhibited the melanoma progression. Moreover, PRDM5 potentiated the progression of murine melanoma via up‐regulating JNK expression.

## Materials and Methods

### Cell culture and reagents

Murine melanoma B16F10 cells and NIH 3T3 cells were purchased from the American Type Culture Collection (ATCC, USA). Cells were routinely cultured in Dulbecco's modified eagle medium (DMEM) supplemented with 10% fetal calf serum (FCS) (Invitrogen, Carlsbad, California, USA) and 1% penicillin/streptomycin (Invitrogen) at 37°C in a humidified atmosphere containing 5% CO_2_. SP600125, a specific inhibitor of JNK was purchased from Sigma (St. Louis, MO).

### Plasmids and DNA constructs

Two sequences specific to mouse *PRDM5* were used to generate siRNAs: target 1 (5’‐GCT GTG CAA TAA GGC CTTT‐3’) and target 2 (5’‐GGA TAC ATT AAA CGT TCAT‐3’). siRNAs and scrambled siRNA were cloned into pSilencer3.1‐U6 and pSilencer4.1‐CMV vectors, respectively, according to the manufacturer's instructions (Ambion, Waltham, Massachusetts, USA). Transfection was carried out with Lipofectamine 2000 (Invitrogen) following the manufacturer's instructions. Cells with stable expression of PRDM5 siRNA were collected after selection with 1 *μ*g/mL puromycin and 800 *μ*g/mL G418. The PRDM5 cDNA was cloned into pXJ‐40‐Myc vector containing a Myc‐tag with specific primers: 5’‐AT GGATCC ATG CTG GGC ATG TAC GTA CCAG‐3’ (forward) and 5’‐AT CTCGAG TTA GCT GTC AGC TAC CCC ATGG‐3’ (reverse).

### Western blotting

Cells were washed twice with cold PBS (3.2 mmol/L Na_2_HPO_4_, 0.5 mmol/L KH_2_PO_4_, 1.3 mmol/L KCl, and 140 mmol/L NaCl, pH 7.4) and lysed in cold lysis buffer (20 mmol/L Tris, 100 mmol/L NaCl, 5 mmol/L EDTA, 1 mmol/L EGTA, 5 mmol/L MgCl_2_, 1% Triton X‐100, 2.5 mmol/L sodium pyrophosphate, 1 mmol/L *β*‐glycerolphosphate, 1 mmol/L Na_3_VO_4_, 1 mmol/L PMSF, and Roche complete protease inhibitors) followed by centrifugation at 15,000*g* for 15 min at 4°C. The supernatant was collected, and protein concentration was determined by Bradford assay (Biorad, Hercules, California, USA). For western blot, proteins were separated by electrophoresis on 8–16% SDS‐PAGE and transferred onto a polyvinylidene fluoride membrane (Millipore, Boston, Massachusetts, USA). After blocking with 5% nonfat milk for 1 h, the membrane was incubated with primary antibodies in 5% nonfat milk (anti‐myc, PRDM5, *β*‐actin, GAPDH [Sigma], p‐AKT, AKT, p‐p38, p38, p‐JNK, JNK, [Cell Signaling Technology]). Visualization was done with Pierce's West Pico chemiluminescence substrate (Millipore) after incubation with horseradish peroxidase (HRP)‐conjugated secondary antibodies.

### Cell proliferation assay

Cell proliferation was determined with Cell Proliferation Kit I (Roche Applied Science, Penzberg, Upper Bavaria, Germany) according to manufacturer's instructions. In brief, 5 × 10^3^ cells were plated into 96‐well plates and grew in complete medium. At different time points, cells were incubated with 3‐[4, 5‐dimethylthiazol‐2‐yl]‐2, 5‐diphenyltetrazoliumbromide (MTT) in fresh medium for 4 h and then with DMSO overnight. Absorbance was measured at 490 nm with Thermo MK3 microplate reader (Thermo Fisher Scientific, Waltham, Massachusetts, USA). At least, three independent experiments were performed in triplicate.

### Wound healing assay

Wound healing assay was performed as previously described 22. Briefly, B16F10 murine melanoma cells were independently seeded into 60‐mm dishes and incubated at 37°C overnight. Then, cells were maintained in a serum‐free medium for 24 h and scratched with a 200‐*μ*L sterile pipette tip to create a wound. The wounded monolayer cells were washed twice with fresh normal medium to remove cell debris. The cell‐free area was photographed at different time points, and the wound healing was evaluated by calculating the percentage of cell‐free area to the initial wounded area.

### In vitro invasion and migration assays

The invasion of melanoma cells was examined by BD BioCoat^TM^ Matrigel^TM^ Invasion Chamber (BD Biosciences, San Jose, California, USA) assay in vitro following the manufacturer's instructions. Briefly, 1 × 10^4^ cells in 100 *μ*L of serum‐free medium were seeded into the upper chamber, and 750 *μ*L of chemo‐attractant, NIH‐3T3 fibroblast‐conditioned medium, was added into the lower chamber. After incubation in a humidified environment with 5% CO_2_ at 37°C for 24 h, the noninvasive cells on the upper surface of the membrane were removed with a cotton swab, and the invasive cells migrating to the lower surface of the membrane were fixed and stained with 0.5% crystal violet for 30 min. Cells migrating to the lower surface were then counted under a light microscope. Ten fields were randomly selected under a microscope at a magnification of 200×. In the migration assay, the chamber membrane was not precoated with matrigel and other procedures were the same to those above.

### Examination of tumor growth and metastasis in vivo

Female C57BL/6J mice aged 6–8 weeks old (15–20 g) were purchased from the Vital River Laboratories (Beijing, China). Protocol and procedures employed were reviewed and approved for handling mice and were approved by the Ethics Committee of Xinxiang Medical University. For examination of tumor growth in vivo, 12 mice were randomly divided into two groups and subcutaneously injected with 1 × 10^6^ B16F10 cells bearing control or PRDM5 siRNA plasmids. Mice were killed 11 days later and the tumor size was determined. For examination of tumor metastasis, single cell suspension (1 × 10^6^) in 100 *μ*L of PBS was injected via the tail vein, and the mice were killed 2 weeks later. All the organs were examined for the macroscopic metastases. Lung metastatic nodules were determined under a dissecting microscope.

### Hematoxylin and eosin staining as well as confocal microscopy

Tumor tissues were cut into 2 mm × 2 mm blocks and then fixed in 4% paraformaldehyde. The tissues blocks were cut into 10‐*μ*m sections and mounted onto poly‐L‐lysine‐coated slides, followed by hematoxylin and eosin (HE) staining. The sections were visualized and photographed using an Olympus IX71 fluorescent microscope (Nikon, Sendai, Japan). For immunohistochemistry, sections were rinsed, blocked with 4% BSA, and incubated with rabbit anti‐PRDM5 antibody (1:1000 in PBS/Triton X‐100) overnight at 4°C. After washing with PBS, sections were incubated with HRP‐conjugated secondary antibody, and nuclei were stained with DAPI. After washing in PBS, sections were mounted with Fluor Save Reagent (Calbiochem, Darmstadt, Germany) and then observed under a confocal microscope (LSM710; Carl Zeiss, Heidenheim, Germany).

### Statistical analysis

Data are expressed in mean ± standard deviation (SD). Statistical analysis was performed using student's *t*‐test or one way analysis of variance (ANOVA) if necessary. A value of *P* < 0.05 was considered statistically significant. SPSS version 20.0 (Armonk, New York, USA) was used for the statistical analysis.

## Results

### PRDM5 overexpression promotes the proliferation, migration, and invasion of B16F10 cells

**Figure 1:**
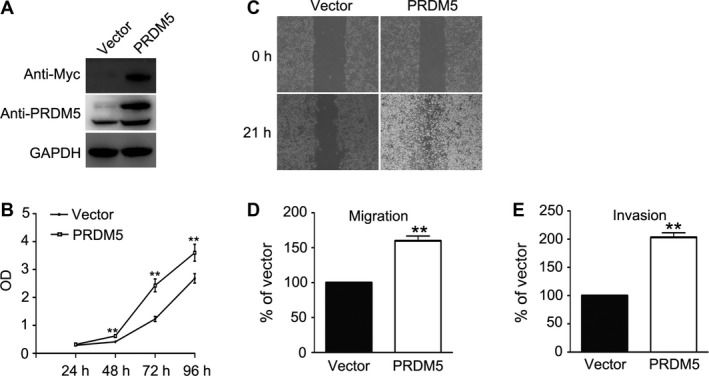
PRDM5 overexpression promotes the proliferation, migration, and invasion of B16F10 cells. (A) PRDM5 was cloned into pXJ‐40 vector and transfected into B16F10 cells. Cells transfected with an empty vector were used as a control. The PRDM5 expression was determined by western blot with antibodies against myc‐tag and PRDM5. GAPDH was used as a loading control. (B) Cell proliferation was determined by 3‐[4, 5‐dimethylthiazol‐2‐yl]‐2, 5‐diphenyltetrazoliumbromide assay. Cell motility was examined by wound healing assay and transwell migration and invasion assays (C, D, E). Data are from three independent experiments and expressed as mean ± SD. **P < 0.01 versus control group.

To assess the role of PRDM5 in the biobehaviors of melanoma, PRDM5 was cloned into pXJ‐40 vector which was then transfected into murine melanoma B16F10 cells. The transfection efficiency was determined by western blot with antibodies against myc‐tag and PRDM5. As shown in Figure 1A, the PRDM5 expression in cells transfected with pXJ‐40‐PRDM5 was greatly increased as compared to those transfected with empty vector. Then, the growth capacity of these cells was investigated by MTT assay and results showed that PRDM5 overexpression significantly enhanced the growth of melanoma cells (Fig. 1B). The effect of PRDM5 overexpression on the migration potential of melanoma cells was also evaluated by wound healing assay. B16F10 cells overexpressing PRDM5 were able to close a wound by 21 h. However, the wound in the control cells had not yet closed at this time point (Fig. 1C). This was further validated by transwell migration and invasion assays (Fig. 1D and E). In summary, PRDM5 overexpression is able to enhance the proliferation and migratory potentials of murine melanoma cells.

### PRDM5 silencing decreases the proliferation, migration, and invasion of B16F10 cells

**Figure 2:**
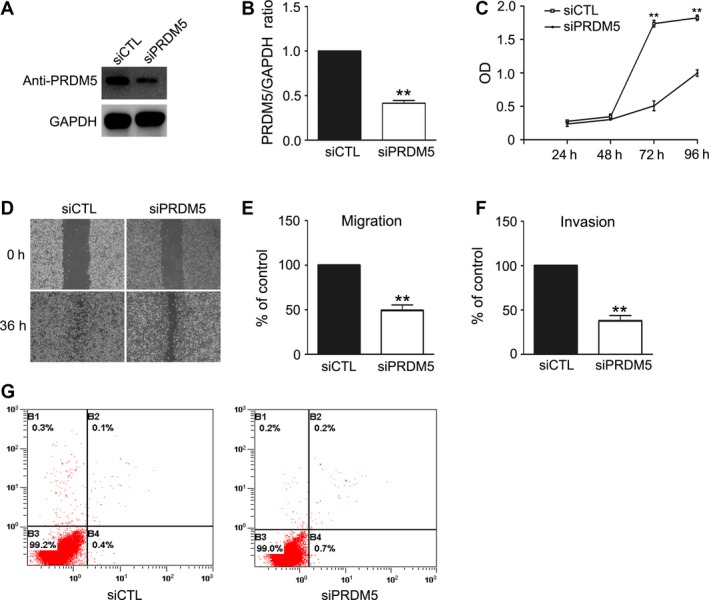
PRDM5 silencing reduces the proliferation, migration, and invasion of B16F10 cells. (A) pSilence3.1‐U6 and pSilence4.1‐CMV vectors carrying siPRDM5 or siCTL were cotransfected into B16F10 melanoma cells separately and stable cell lines were generated by selection with G418 and puromycin. PRDM5 expression was assessed by western blot. GAPDH was used as a loading control. (B) Level of PRDM5 was quantified by densitometry and normalized to GAPDH. (C) Cell proliferation was determined by 3‐[4, 5‐dimethylthiazol‐2‐yl]‐2, 5‐diphenyltetrazoliumbromide assay. Cell motility was examined by wound healing assay and transwell cell migration and invasion assays (D, E, F). Data are from three repeated experiments and expressed as mean ± SD. **P < 0.01 versus control group. (F) Flow cytometry was performed to determine the apoptotic cells in siCTL and siPRDM5 groups.

To further confirm the function of PRDM5 in the proliferation, migration, and invasion of melanoma cells, its expression was down‐regulated with a siRNA‐mediated approach in murine melanoma B16F10 cells. Significant silencing was achieved by transfecting B16F10 cells with pSilencer3.1‐U6 and pSilencer4.1‐CMV‐based plasmid‐expressing siRNAs targeting different regions of mouse *PRDM5* mRNA. B16F10 cells transfected with scrambled siRNA without sequence homology to any known mouse gene were used as control. PRDM5 expression in cells transfected with siRNA plasmids (siPRDM5) was significantly reduced as compared to that in cells transfected with scrambled siRNA (siCTL) as determined by western blot (Fig. 2A and B). The effect of PRDM5 gene silencing on cell growth was then examined by MTT assay. As shown in Figure 2C, PRDM5 silencing significantly reduced the growth of melanoma cells. Next, wound healing assay and transwell migration and invasion assays were employed to examine the influence of PRDM5 gene silencing on the migration and invasion of melanoma cells. As expected, migration and invasion potentials of cells with PRDM5 gene silencing were significantly decreased when compared with cells transfected with scrambled siRNA (Fig. 2D, E and F). To exclude the possibility that PRDM5 gene silencing increase cell vulnerability, thus affecting cell growth and metastasis, apoptotic cell death was evaluated by flow cytometry. Results showed no significant difference in the apoptotic cells between siCTL group and siPRDM5 group (Fig. 2G). In conclusion, PRDM5 silencing significantly reduced the proliferation, migration, and invasion potentials of murine melanoma cells.

### PRDM5 silencing inhibits the growth and metastasis of melanoma in vivo

**Figure 3:**
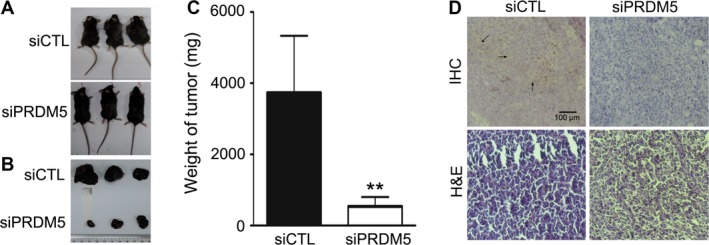
PRDM5 silencing inhibits the melanoma growth in vivo. (A) Mice at 11 days after inoculation of siCTL or siPRDM5 cells. (B) Specimen of melanoma tumors. (C) Quantification of the tumor weight. **P < 0.01 versus control group. (D) Immunohistochemistry for PRDM5 expression in melanoma. Scale bars: 100 *μ*m.

To explore the role of PRDM5 in the growth of melanoma in vivo, B16F10 cells transfected with siPRDM5 or control plasmids were subcutaneously inoculated into C57BL/6J mice. On day 11, the tumor size in siPRDM5 group was significantly smaller than in siCTL group (Fig. 3A and B) and the tumor weight in siPRDM5 group was also significantly lighter than in siCTL group (Fig. 3C), indicating that PRDM5 silencing inhibits the growth of melanoma cells in vivo. Moreover, confocal microscopy confirmed that PRDM5 expression in siPRDM5 group was significantly lower than in siCTL group (Fig. 3D, upper panels). Interestingly, the melanoma in siPRDM5 group also exhibited more compacted growth than in siCTL group as shown by HE staining (Fig. 3D, lower panels).

**Figure 4:**
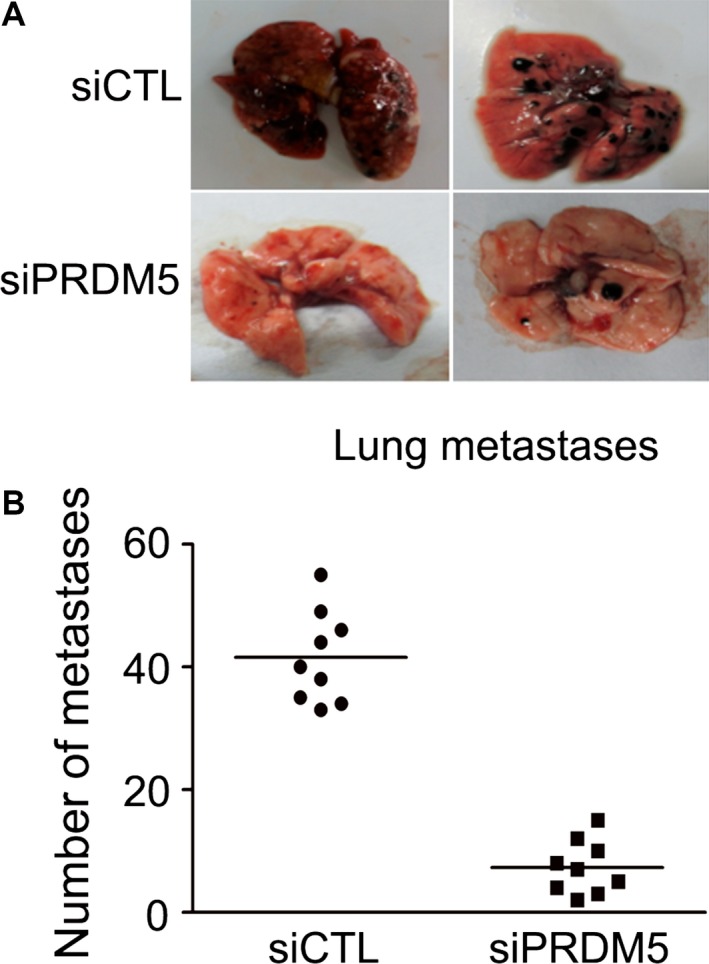
PRDM5 silencing inhibits melanoma metastasis in vivo. (A) Lungs from mice inoculated with siCTL or siPRDM5 cells were collected at 14 days after cell injection. (B) Metastatic foci in the lungs were quantified at different time points, **P < 0.01, versus control group.

To examine the effect of PRDM5 on the metastasis of melanoma in vivo*,* experimental metastasis assay of melanoma was performed. Fourteen days after tumor cell inoculation, mice were killed and all the organs were collected for examination of metastatic foci. As shown in Figure 4A, metastatic foci were mainly observed in the lungs as previously reported 23. Compared with the control group, the number of metastatic foci was significantly reduced in mice injected with PRDM5 knock‐down cells (Fig. 4B), suggesting that PRDM5 silencing impairs the metastatic potential of melanoma cells in vivo.

### JNK pathway is involved in PRDM5‐mediated progression of murine melanoma cells

**Figure 5:**
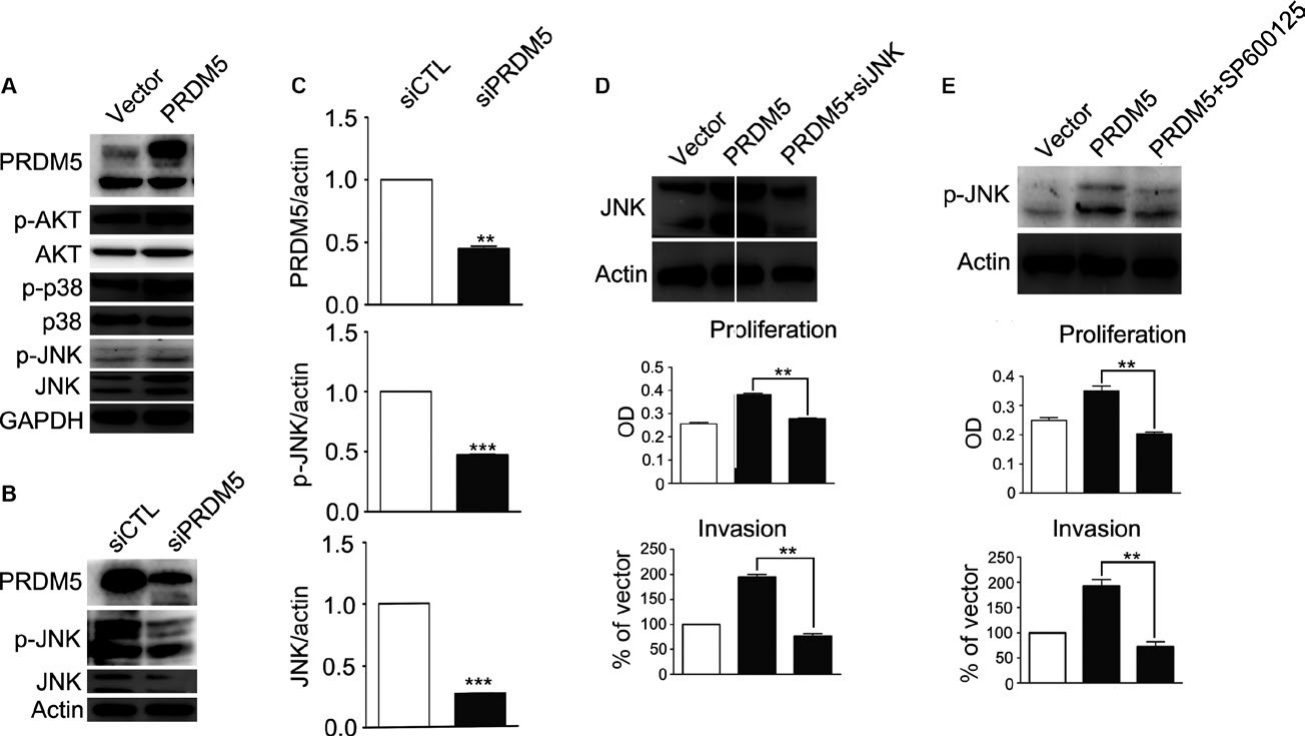
JNK pathway is involved in PRDM5‐mediated proliferation of murine melanoma cells. (A) Western blot showed that the expression of JNK and phosphorylated JNK was elevated in cells transfected with pXJ‐40‐PRDM5. GAPDH was used as a loading control. (B) The expression of JNK and phosphorylated JNK was inhibited in cells transfected with PRDM5 siRNA. Actin was used as a loading control. (C) Levels of PRDM5, p‐JNK, and JNK were quantified by densitometry and normalized to actin. (D) Western blot shows that PRDM5‐induced JNK expression was inhibited by JNK siRNA. Actin was used as a loading control. MTT assay and transwell invasion assay were performed to determine the proliferation and invasiveness of cells cotransfected with pXJ‐40‐PRDM5 and JNK siRNA. (E) Cells were incubated with SP600125 for 1 h, and total protein was extracted for western blot with antibodies against phosphorylated JNK or actin. MTT assay and transwell invasion assay were performed to determine the proliferation and motility of cells transfected with pXJ‐40‐PRDM5 after SP600125 treatment. *P<0.05, versus control group. **P<0.01 versus control group. ***P<0.001 versus control group. MTT, 3‐[4, 5‐dimethylthiazol‐2‐yl]‐2, 5‐diphenyltetrazoliumbromide.

To determine the signaling pathways involved in PRDM5‐mediated progression of melanoma cells, signaling pathways related to melanoma progression were examined. As shown in Figure 5A, the JNK expression and activation significantly increased in cells overexpressing PRDM5, but the expression and activation of AKT and p38 remained unchanged. Consistently, when PRDM5 expression was silenced by siRNA in melanoma cells, JNK expression and activation were also reduced (Fig. 5B and C). This suggests that PRDM5 is able to increase the expression and activation of JNK in B16F10 cells.

To explore the role of JNK in the PRDM5‐mediated proliferation and invasion of B16F10 cells, JNK expression was silenced in PRDM5‐overexpressing cells with a commercial JNK siRNA. As expected, JNK silencing significantly reduced PRDM5‐mediated up‐regulation of JNK expression and blocked the PRDM5‐induced proliferation and invasion of B16F10 cells (Fig. 5D). To further verify the involvement of JNK signaling in PRDM5‐induced progression of melanoma, SP600125, a specific JNK inhibitor, was employed to inhibit the JNK signaling pathway. As shown in Figure 5E, PRDM5‐mediated JNK activation was completely blocked by SP600125. As a result, PRDM5‐induced proliferation and invasion in B16F10 cells were also abolished by SP600125, as revealed by MTT assay and transwell invasion assay (Fig. 5E). These findings indicate that JNK signaling pathway is involved in PRDM5‐induced progression of melanoma.

## Discussion

PRDM5 is a transcription factor of the PR domain protein family characterized by the presence of a PR domain and multiple zinc finger motifs. Members of the PR domain family are reported to be involved in cell differentiation and tumorogenesis 24. However, no study has been conducted to investigate the role of PRDM5 in the pathogenesis of melanoma. In this study, we determined the function of PRDM5 in the pathogenesis of melanoma. Of interest, our results showed that PRDM5 overexpression significantly enhanced the proliferation, migration, and invasion of murine melanoma B16F10 cells, and silencing of PRDM5 expression was found to reduce the proliferation and metastasis of these cells. Furthermore, the cancer growth and metastasis were significantly inhibited in mice inoculated with cells undergoing PRDM5 silencing in vivo. These results indicate that PRDM5 functions as a potential oncogene in murine melanoma. Of note, we further evaluated the effects of PRDM5 on the in vitro proliferation and migration of human melanoma HTB‐72 cells and murine melanoma B16F0 cells. Convincingly, results also showed that PRDM5 overexpression significantly enhanced the proliferation and migration of HTB‐72 cells (Fig. S1) and B16F0 cells (Fig. S2). This indicates that the effects of PRDM5 in melanoma do not vary between species.

Surprisingly, the role of PRDM5 in melanoma is different from that reported in other studies. Generally, PRDM5 acts as a tumor suppressor in most cancers. It has been reported that PRDM5 expression is reduced in human breast cancer, ovarian cancer, and liver cancer, and reintroduction of PRDM5 into ovarian cancer cells causes G2/M cell cycle arrest and apoptotic cell death 14. In addition, PRDM5 expression at both mRNA and protein levels has been found to be down‐regulated in lung squamous cell carcinoma due to aberrant cytosine methylation of the promoter of PRDM5 gene, and treatment with a DNA methyltransferase inhibitor reactivates PRDM5 expression accompanied by the decreased proliferation of lung squamous cell carcinoma cells 19. Moreover, PRDM5 expression is undetectable in colorectal cancer and gastric cancer due to DNA methylation or trimethylation of Lys27 of histone H3 (H3K27) and PRDM5 overexpression significantly inhibits the colony formation of gastric cancer cells 20. The reason why PRDM5 works in opposite directions between melanoma and epithelial tumors, such as breast cancer, ovarian cancer, and liver cancer, may be ascribed to its unique function in transcription regulation. It has been reported that PRDM5 can either promote or suppress gene transcription depending on the promoter and cell types 25. PRDM5 might activate a set of genes favoring cell proliferation and migration in melanoma cells, whereas it inactivates another set of genes in epithelial tumors.

Although our study for the first time showed that PRDM5 acts as an important promoter in the progression of malignant melanoma, it is unclear how PRDM5 works.

The underlying molecular mechanism for PRDM5‐regulated melanoma progression was identified to be related to JNK signaling pathway. Our results showed that PRDM5 overexpression in B16F10 murine melanoma cells induced up‐regulation of JNK expression, and PRDM5 silencing reduced JNK expression. In addition, JNK silencing or JNK inhibitor could block the enhanced proliferation, migration, and invasion of B16F10 cells secondary to PRDM5 overexpression. The involvement of JNK in cancer progression can be supported by the fact that suppression of JNK activation by tumor suppressor cylindromatosis (CYLD) inhibits melanoma progression 19. In addition, another tumor suppressor, p16/INK4a, inhibits UV‐induced malignant transformation of melanoma by interacting with JNK or suppressing JNK activation 26. Moreover, JNK has also been reported to mediate CD‐9‐promoted transendothelial invasion of melanoma by enhancing synthesis of MMP‐2 27. It is difficult to precisely determine how PRDM5 up‐regulates the JNK expression. We speculated that the mechanism underlying this specificity involves the ability of PRDM5 binding to the transcriptional start site (TSS) or gene bodies of its target genes. It has been reported that PRDM5 stimulates collagen I transcription by binding the *Col1a1* gene body and maintaining the RNA polymerase II occupancy 4. In the future, we will perform chromatin immunoprecipitation (ChIP) and DNA sequencing to determine whether PRDM5 directly interacts with TSS or gene body of JNK to up‐regulate its expression.

In summary, our results indicated that PRDM5 potentiates the progression of murine melanoma through up‐regulating JNK expression, indicating that PRDM5 may function as an oncogene in melanoma. Due to the important roles of PRDM5 in the progression of melanoma, it will become an attractive target for the molecular therapy of melanoma. In the future, it is worthwhile to delineate the specific mechanism underlying PRDM5‐mediated JNK expression as well as PRDM5‐induced progression of melanoma.

## Conflict of Interest

The authors have declared that no competing interest exists.

## Supporting information


**Figure S1.** PRDM5 overexpression promotes the proliferation and migration of human melanoma HTB‐72 cells. (A) Human melanoma HTB‐72 cells were transfected with plasmid expressing PRDM5, and an empty vector was used as a control. The PRDM5 expression was determined by western blotting with antibody against PRDM5. The proliferation (B) and migration (C) of these cells were determined by MTT assay and transwell migration assay, respectively. *P < 0.05, **P < 0.01 versus empty vector group.

**Figure S2.** PRDM5 overexpression promotes the proliferation and migration of murine melanoma B16F0 cells. (A) Murine melanoma B16F0 cells were transfected with plasmid expressing PRDM5, and an empty vector was used as a control. The PRDM5 expression was determined by western blotting with antibody against PRDM5. The proliferation (B) and migration (C) of these cells were determined by MTT assay and transwell migration assay, respectively. *P < 0.05, **P < 0.01, ***P < 0.001 versus empty vector group.
