# Supplementary figures and images for: PRDM5 promotes the proliferation and invasion of murine melanoma cells through up‐regulating JNK expression
Source: Cancer Med. 2016 Aug 3;5(9):2558–66. doi: 10.1002/cam4.846 (PMC5055150; doi:10.1002/cam4.846)

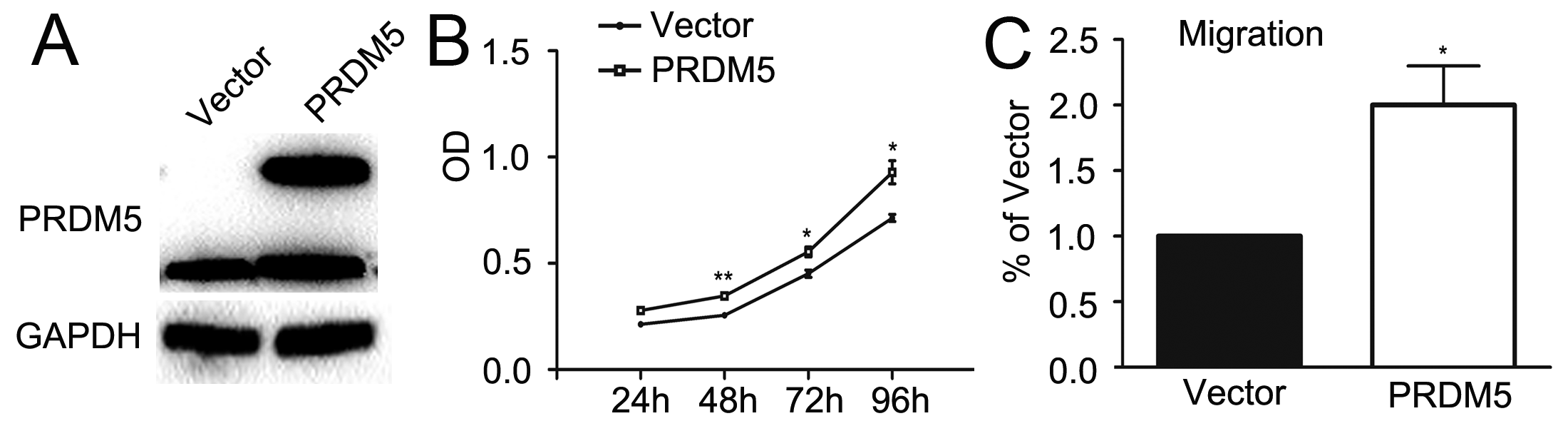

Supplement: Supplementary file 1 — Figure S1. PRDM5 overexpression promotes the proliferation and migration of human melanoma HTB‐72 cells. (A) Human melanoma HTB‐72 cells were transfected with plasmid expressing PRDM5, and an empty vector was used as a control. The PRDM5 expression was determined by western blotting with antibody against PRDM5. The proliferation (B) and migration (C) of these cells were determined by MTT assay and transwell migration assay, respectively. *P < 0.05, **P < 0.01 versus empty vector group. [file CAM4-5-2558-s001.tif]

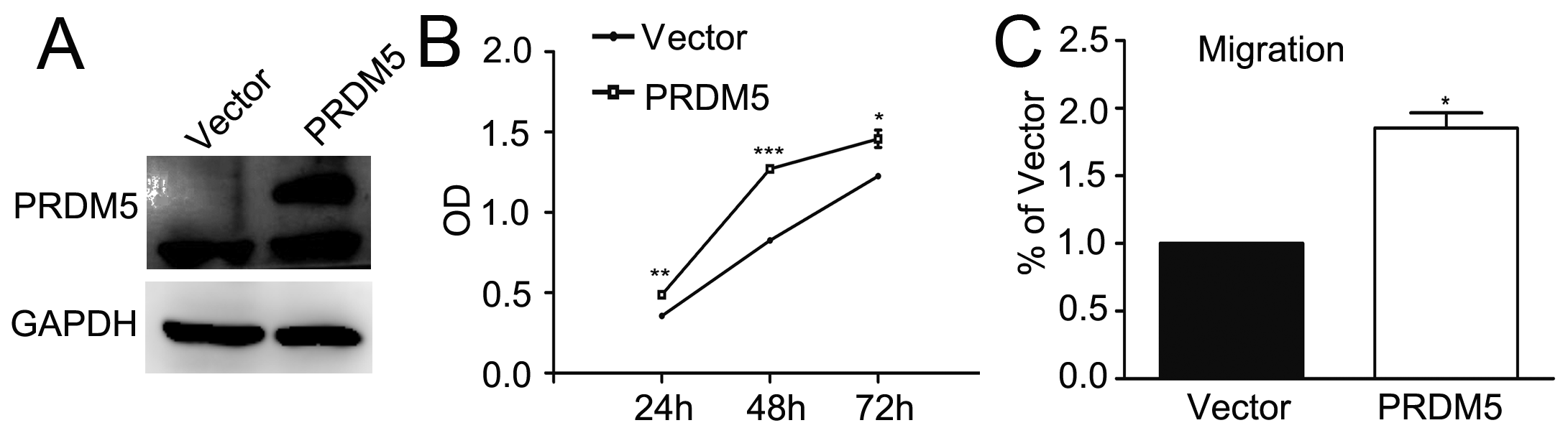

Supplement: Supplementary file 2 — Figure S2. PRDM5 overexpression promotes the proliferation and migration of murine melanoma B16F0 cells. (A) Murine melanoma B16F0 cells were transfected with plasmid expressing PRDM5, and an empty vector was used as a control. The PRDM5 expression was determined by western blotting with antibody against PRDM5. The proliferation (B) and migration (C) of these cells were determined by MTT assay and transwell migration assay, respectively. *P < 0.05, **P < 0.01, ***P < 0.001 versus empty vector group. [file CAM4-5-2558-s002.tif]
